# Double-strand break-free epigenetic programming: a safer path for T-cell therapies

**DOI:** 10.1038/s41392-025-02529-9

**Published:** 2026-01-12

**Authors:** Lanxin Deng, Yujia Yang, Assam El-Osta

**Affiliations:** 1 Baker Heart and Diabetes Institute, Epigenetics in Human Health and Disease Program, Melbourne, Vic Australia; 2 Baker Department of Cardiometabolic Health, The University of Melbourne, Parkville, Vic Australia; 3 School of Translational Medicine, Department of Diabetes, Monash University, Melbourne, Vic Australia; 4 Department of Medicine and Therapeutics, The Chinese University of Hong Kong (CUHK), Hong Kong SAR, China

**Keywords:** Genetic engineering, Molecular medicine

In a recent study published by *Nature Biotechnology*,^1^ Goudy et al. described an all-RNA CRISPRoff/CRISPRon platform that programs endogenous gene expression in primary human T-cells without introducing double-strand breaks, offering a solution to the long-standing safety limitations of nuclease-based multiplex editing. This platform demonstrates durable and locus-specific gene silencing or activation across multiple targets, improved in vivo tumor control, and a scalable, low-toxicity path for next-generation T-cell engineering.

The scientific rationale is straightforward: DSBs can activate p53-mediated stress responses in human cells.^2^ Epigenetic editors avoid these liabilities by binding without cleaving, installing reversible chromatin states instead of permanent sequence changes. This design has two advantages. First, durability with reversibility: silencing can persist through proliferation and repeated stimulation yet can be lifted if needed. Epigenetic memory has moved from hypothesis to capability: CRISPR off‑driven silencing can persist through many divisions, repeated antigen stimulation, and even after in vivo transfer; CRISPR on can reactivate the locus when needed. Functionally, these findings support designs where T‑cell exhaustion pathways are disrupted during expansion and early tumor engagement and then restored to mitigate long‑term risks or where lineage‑defining programs such as Treg‑like phenotypes induced by FOXP3 TSDR demethylation are stabilized in a controlled manner. This tunability goes beyond on/off control, enabling dose-dependent and guide-number-dependent expression levels. The second is scale: because toxicity from simultaneous DSBs is avoided, multiple guides can be delivered to coordinate complex phenotypes. In side‑by‑side comparisons, multiplex epigenetic silencing achieved high efficiency with minimal cell loss, whereas triple–quintuple nuclease knockouts were deleterious to T‑cell fitness. Because the underlying sequence remains intact, expression can be graded by guide number and dose, mirroring natural gene regulation rather than enforcing a static knockout. This gives clinicians a dimmer switch, not just an on/off knife.

Dispense with the myth that CRISPR off works only on CpG island promoters. CpG islands are receptive; they do not define the addressable genome. However, durable repression has been demonstrated at many non‑island genes; performance is gene‑specific but tunable by guide pooling and dose. Examples include robust silencing of inhibitory receptors and checkpoint genes over several weeks, with partial but adjustable effects at more refractory loci, broadening the targetable space beyond classic island promoters.^3^

Another virtue is orthogonality. A single, justified knock-in—say, inserting a CAR into TRAC—can be layered with epigenetic programming on top. As shown in the present study, TRAC knock-in (AsCas12a) combined with CRISPRoff-mediated silencing of RASA2 increased in vivo tumor control without loss of knock-in efficiency; sustained CD151 repression post-transfer documented epigenetic memory.^1^ This “do both” pattern keeps potency high and structural-variant risk in check in the same manufacturing run.

In practice, applying these editing principles in manufacturing will require scalable processes. mRNA electroporation fits standard GMP, avoids integration, and minimizes editor persistence. In previous work showing that RASA2 loss enhances CAR-T-cell persistence and effector function, CRISPRoff-mediated RASA2 silencing in TRAC-KI CAR-T-cells improved in vivo control and survival, whereas CD151 silencing remained intact post-transfer, indicating durable epigenetic memory through proliferation.^4^ The practical pitch is straightforward: reserve the rare DNA cut for adding a new receptor or safe harboring a payload; do the rest with programmable chromatin.

Moving from permanent edits to reversible programming does not erase risk—it redefines it. Regulatory readiness depends on several interrelated domains: epigenetic specificity, genomic stability, immune recognition of the editor, and control over the durability of memory. Epigenetic off-target effects remain a concern since dCas9 fusions can introduce marks at unintended loci; ChIP-seq or CUT&RUN combined with locus-resolved bisulfite or oxidative bisulfite sequencing can demonstrate on-target engagement and methylation specificity, whereas long-read platforms such as the Oxford Nanopore or PacBio can be incorporated into lot-release panels to detect translocations and large structural variants associated with knock-ins. In parallel, both innate and adaptive immune responses to the editor and its RNA should be characterized, and the persistence of the protein reported relative to the durability of its effect, with inducible or erasable modules—such as drug-responsive dCas9-TET1 constructs—providing a reversible safety mechanism when durable programming is no longer desired.

**Fig. 1:**
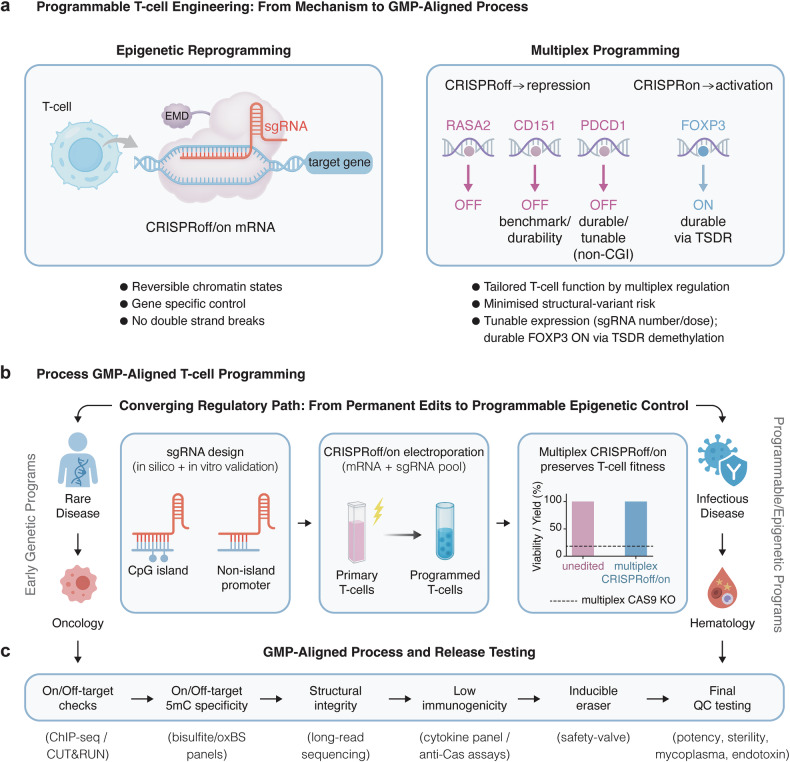
Programmable T-cell engineering from an epigenetic mechanism to a GMP-aligned process. **a** Epigenetic reprogramming in primary human T-cells via transient delivery of dCas9-CRISPRoff/CRISPRon mRNA with sequence-specific sgRNAs, enabling reversible chromatin rewriting without double-strand breaks. EMD (epigenetic modifier domain). Multiplex CRISPRoff installs repressive chromatin states to silence functional targets (RASA2), benchmark durability loci (CD151), and non-CpG-island checkpoint genes such as PDCD1/PD-1, achieving repression that is durable yet tunable. In parallel, CRISPRon enables durable gene activation through targeted enhancer demethylation (illustrated by FOXP3 ON via TSDR demethylation). Tunability is achieved by selecting the sgRNA, number, and dose, enabling adjustable regulatory set-points. **b** GMP-aligned programming workflow showing (i) sgRNA design and selection across CpG island and non-island promoter contexts with in silico and in vitro validation, (ii) single-step electroporation of the editor mRNA + multiplex sgRNA pool into primary T-cells, producing programmed T-cells while preserving yield/viability relative to multiplex nuclease KO, and (iii) a converging regulatory trajectory from early permanent genetic programs (rare disease, oncology) toward programmable epigenetic programs (infectious disease, hematology). An optional single TRAC knock-in using an orthogonal nuclease, such as Cas12a, may be performed during the electroporation step but is not shown here. **c** Release testing for GMP readiness verifies on- and off-target chromatin remodeling (ChIP-seq/CUT&RUN), 5-methylcytosine specificity and durability (bisulfite/oxBS panels), structural integrity and SV risk (long-read sequencing), low immunogenicity (cytokine profiling and anti-Cas assays), functionality of an inducible eraser/safety-valve module, and final QC including identity, potency, sterility, mycoplasma, and endotoxin

CRISPR-based cell therapies have reached key regulatory milestones. CASGEVY (exagamglogene autotemcel; exa-cel) is FDA-approved for sickle cell disease (SCD) in patients ≥12 years with recurrent vaso-occlusive crises (8 Dec 2023) and for transfusion-dependent β-thalassaemia (TDT; 16 Jan 2024). In the UK, the Medicines and Healthcare products Regulatory Agency (MHRA) authorized CASGEVY in November 2023, and the National Institute for Health and Care Excellence (NICE) issued managed-access technology appraisals—TA1003 for TDT (11 Sep 2024) and TA1044 for SCD (26 Feb 2025)—supporting NHS adoption. These approvals are grounded in pivotal excel trials, CLIMB SCD-121 (NCT03745287) and CLIMB THAL-111 (NCT03655678), with results published in *N Engl J Med* (2023). CRISPR-edited T-cell programs continue: CTX112 is recruiting for B-cell malignancies (NCT05643742) and autoimmune indications (NCT06925542); CB-010 is still recruiting in the ANTLER phase 1 trial (NCT04637763), whereas CTX110 (NCT04035434) has been terminated with participants in long-term follow-up. In addition to T-cells, a first-in-human study published in *N Engl J Med* demonstrated that gene-edited ‘hypoimmune’ allogeneic β cells survived and functioned without immunosuppression, highlighting how cell-intrinsic programming can reshape risk–benefit profiles for living drugs.^5^ Collectively, these programs—predominantly nuclease-edited today, with epigenetic programming emerging—trace a trajectory from correction to programmable states, supported by GMP-aligned workflows and standardized specificity assays (Fig. 1).

Epigenetic CRISPR systems provide a new framework for T‑cell engineering. Rather than making permanent genetic edits, they enable tunable expression states. If paired with regulator-ready analytics and thoughtful product design—cut sparingly, program frequently—the next chapter of cell therapy will be driven not by the blade but by the dial.
